# Free and Bound Phenolic Profiles and Antioxidant Activities in Melon (*Cucumis melo* L.) Pulp: Comparative Study on Six Widely Consumed Varieties Planted in Hainan Province

**DOI:** 10.3390/foods12244446

**Published:** 2023-12-12

**Authors:** Yuxi Wang, Heqi Gao, Zhiqiang Guo, Ziting Peng, Shuyi Li, Zhenzhou Zhu, Nabil Grimi, Juan Xiao

**Affiliations:** 1 Hainan Engineering Research Center of Aquatic Resources Efficient Utilization in South China Sea, Key Laboratory of Seafood Processing of Haikou School of Food Science and Engineering, Hainan University, Haikou 570228, China; 2 School of Marine Science and Engineering, Hainan University, Haikou 570228, China; 3 National R&D Center for Se-Rich Agricultural Products Processing, Hubei Engineering Research Center for Deep Processing of Green Se-Rich Agricultural Products, School of Modern Industry for Selenium Science and Engineering, Wuhan Polytechnic University, Wuhan 430023, China; shuyi.li@whpu.edu.cn (S.L.);; 4 Centre de Recherche Royallieu, Université de Technologie de Compiègne, Sorbonne Universités, CS 60319, 60203 Compiègne CEDEX, France

**Keywords:** melon pulp, bound phenolic compound, free phenolic compound

## Abstract

Bound phenolic compounds in the melon pulp have seldom been investigated. This study revealed considerable differences in the total phenolic content (TPC) and antioxidant activity of the free and bound phenolic extracts in the pulps of six melon varieties from Hainan Province, China. Naixiangmi and Yugu demonstrated the highest free TPC, while Meilong showed the highest bound and total TPC and antioxidant activity. UHPLC-QQQ-MS identified and quantified 30 phenolic compounds. The melon cultivars markedly differed in the amount and content of their free and bound phenolic compounds. Xizhoumi No. 25 and Meilong afforded the most phenolic compounds. Hongguan emerged with the highest free phenolic compound content and total content of phenolic compounds; however, Meilong possessed the highest bound phenolic compound content. Hierarchical cluster analysis divided the melon varieties into four different taxa. The present study provides a scientific basis for developing the health-promoting effects of melon pulp.

## 1. Introduction

Melon (*Cucumis melo* L.; Order Cucurbitales; Family Cucurbitaceae) has a high commercial value and is widely cultivated throughout Europe, Africa, South America, and Asia. Global melon production (including cantaloupe) was 28.47 million tons in 2020 [1]. Melon is favored by consumers because of its pleasant aroma, taste, and nutritional and medicinal properties [2]. Melon pulp is rich in vitamins and dietary fiber, as well as low in fat and calories [3]. It has been used to alleviate pain and as a diuretic in traditional Chinese medicine [2]. It is also an indispensable source of phytochemicals, for instance, phenolics and β-carotene, which have potent antioxidant activity [3]. These plant secondary metabolites prevent chronic diseases, including cardiovascular disease, inflammation, and certain types of cancer [2,4,5].

Phenolics are ubiquitous phytochemicals. They may occur in free or bound form, depending on their association with the food matrix. Free phenolics are localized in vacuoles, easily extracted by solvents and absorbed in the small intestine. By contrast, bound phenolics are structurally connected to cellulose and other polysaccharides, usually extractable by hydrolysis, and are only slightly absorbed in the small intestine [5]. Hence, the nutritional value of bound phenolics has attracted very little research attention. Nevertheless, *in vitro*, bound phenolics extracted by hydrolysis demonstrated strong antioxidant and anti-inflammatory activity [5]. *In vivo*, the bound phenolics exert strong physiological effects after being released from the cell wall components and metabolizing by the colonic microbiota [6]. The majority of recent studies have focused on the diversity of total phenolic content (TPC) and the antioxidant ability of free phenolic extracts of melon pulp [3,7,8,9]. It also partially identified free phenolic compounds by ultra-high performance liquid chromatography-tandem mass spectrometry and high-performance liquid chromatography [2,10]. However, there is comparatively little knowledge about the profile of bound phenolic compounds in the melon pulp. An earlier study reported the TPC and antioxidant capability of bound extracts acquired from three melon cultivars cultivated in Japan [9]. Thus, in-depth investigations into bound phenolic compounds in melon pulp are necessary.

China is the world’s leading producer of melons, accounting for 48.71% of global melon production [1]. Hainan, Xinjiang, and Gansu are the main melon-producing areas of China. Nevertheless, Hainan has a tropical monsoon climate with abundant sunshine, high temperature, and short crop cycles. Therefore, high-quality melon can be planted and harvested year-round in Hainan [11]. Previous studies reported abundant free phenolics in the pulps of melons cultivated in India, Malaysia, Egypt, and Brazil [7,10]. On the other side, comparatively little research has been conducted focusing on the phenolic compounds in Chinese melon pulp. Furthermore, these studies targeted mainly melon produced in Xinjiang. To our best knowledge, no prior investigations into the free and bound phenolic profiles of melon from Hainan Province have been performed.

Here, free and bound phenolics were extracted from the pulp of six popular melon varieties planted in Hainan Province and evaluated the TPC and antioxidant ability. Individual phenolic compounds in free and bound phenolic extracts were characterized and quantified by ultrahigh-performance liquid chromatography-Xevo triple quadrupole mass spectrometry (UHPLC-QQQ-MS). The phenolic profiles of the six melon varieties were also compared by principal component analysis (PCA) and hierarchical cluster analysis (HCA).

## 2. Materials and Methods

### 2.1. Materials and Reagents

Fresh melons (*Cucumis melo* ssp. *melo*) (Figure S1) at the commercial maturity stage and free of obvious traumatic injury were purchased from the North and South Fruit Market, Haikou, Hainan Province, China in November 2021. The varieties were Xizhoumi No. 25, Xiaomi No. 25, Naixiangmi, Hongguan, Yugu, and Meilong. All samples were rinsed with deionized water to remove visible dust, wiped dry with dust-free tissues, and separated into pulp, seeds, and peel. The pulp was then cut into 2 cm^3^ cubes, lyophilized, ground, randomly mixed, and stored at −20 °C.

All chemical reagents used in the UHPLC-QQQ-MS were HPLC grade while all others were analytical grade. Total antioxidant capacity assay kits were purchased from Nanjing Jiancheng Bioengineering Institute (Nanjing, China) and were based on the ferric-reducing antioxidant power (FRAP) and 2,2′-azino-bis(3-ethylbenzthiazoline)-6-sulfonic acid (ABTS) radical scavenging activity analytical methods.

### 2.2. Extraction of Free Phenolic Compounds from Melon Pulp

Free phenolic compounds were extracted from the melon pulp on the basis of a slightly modified method formerly reported [12]. After 20 mL 70% (*v*/*v*) ethanol was added to the melon pulp powder (1 g), the mixture was homogenized in an XHFD homogenizer (IKA-Labortechnik, Staufen, Germany) at 8000 rpm for 5 min at 4 °C, and then centrifuged at 10,000 rpm for 15 min at 4 °C. Residues were repeatedly extracted in a preceding manner. The supernatants of two consecutive extractions were vacuum-concentrated to dryness at 45 °C, redissolved in 7 mL of 85% (*v*/*v*) aqueous methanol, and stored at −20 °C.

### 2.3. Extraction of Bound Phenolic Compounds from Melon Pulp

As previously reported, the alkaline hydrolysis method was utilized to extract the bound phenolic compound [12]. The remaining residue following the extraction of the free phenolic compounds was mixed with 40 mL of 2 M NaOH, subjected to an N_2_ stream for 5 min, and continuously shaken to hydrolyze for 18 h at room temperature. The mixtures were centrifuged at 10,000 rpm and 4 °C for 15 min and the precipitates were repeatedly hydrolyzed. The supernatants of two hydrolysates were collected, adjusted to pH 1–2, and extracted six times with an equal volume of ethyl acetate as the supernatant. Finally, the ethyl acetate fractions were concentrated to dryness under vacuum at 45 °C, redissolved in 7 mL of 85% (*v*/*v*) aqueous methanol, and preserved at −20 °C.

### 2.4. TPC Determination

The total phenolic content (TPC) of free and bound phenolic extracts was measured by the Folin–Ciocalteu colorimetric method at 765 nm, using gallic acid as the standard [12]. Briefly, the Folin–Ciocalteu reagent was mixed with the diluted extract, and incubated for 6 min, followed by the addition of Na_2_CO_3_ solution (7%, *m*/*v*). The mixture was incubated in the dark for 1 h and then measured using a RT-6100 microplate reader (Rayto Life and Analytical Sciences Co., Ltd., Shenzhen, China). The TPC was presented as milligrams of gallic acid equivalents per hundred grams of dry weight (mg GAE/100 g DW) of melon pulp powder. Total TPC was the addition of free and bound TPC.

### 2.5. Phenolic Compound Identification and Quantification by UHPLC-QQQ-MS

Based upon a previously reported method [12], identification and quantification of individual free and bound phenolic compounds were conducted by UHPLC-QQQ-MS (Waters Corp., Milford, MA, USA) combined with multiple reaction monitoring (MRM). A Waters BEH-C18 column (1.7 μm; 2.1 mm i.d. × 100 mm length) was used for separating the phenolic compounds. Mobile phases A and B consisted of 0.5% (*v*/*v*) formic acid-water and acetonitrile, respectively. The separation gradient program was 5% B at 0–1 min, 25% B at 8 min, 60% B at 12 min, 100% B at 13–16 min, and 5% B at 16.1–18 min. The phenolic compounds were tentatively characterized by comparisons with their parent and fragment ions against MS data reported in the literature. The phenolic compounds were assigned with UHPLC-QQQ-MS by comparing them against phenolic standards with similar basic structures. They were quantified in μg/g DW using calibration curves of their respective standards. The parameters were mass range = 100–1000, capillary voltages = 5.5 kV (negative mode) and 4.5 kV (positive mode), drying gas = N_2_ flow at 1000 L/h, and drying gas temperature = 500 °C.

### 2.6. Antioxidant Capacity

Commercial FRAP and ABTS^+^ assay kits were utilized to determine antioxidant capacity following the manufacturer’s protocol. For FRAP assay, the diluted extract was mixed with fresh FRAP working solution, reacted for 30 min in dark at room temperature, and determined using a RT-6100 microplate reader at 593 nm. For ABTS^+^ assay, the diluted extract was mixed with ABTS^+^ working solution, reacted for 6 min at room temperature, and determined using a RT-6100 microplate reader at 734 nm. Comparisons of the phenolic extracts against standard FeSO_4_ and Trolox curves yielded antioxidant capacity values that were calculated and expressed in mM Fe(II)E/g DW for FRAP and mM TE/g DW for ABTS^+^. Total antioxidant activity was the addition of free and bound antioxidant activity.

### 2.7. Statistical Analysis

Data are presented as means ± standard deviation (SD). All determinations were repeated at least thrice. One-way analysis of variance (ANOVA) followed by Duncan’s post hoc test was performed in SPSS Statistics v.26.0 (IBM, Armonk, NY, USA) to identify significant differences between treatments. Differences were considered statistically significant at *p* < 0.05. Principal component analysis (PCA), hierarchical clustering analyses (HCA), and correlation plots were performed in Origin 2021 (OriginLab, Northampton, MA, USA).

## 3. Results and Discussion

### 3.1. TPC

**Figure 1:**
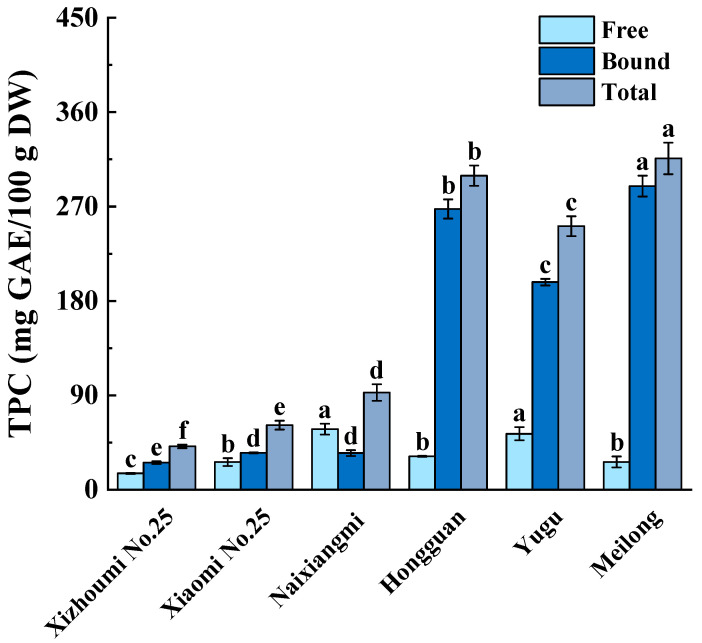
Free, bound, and total TPC in the pulp of six melon varieties. Different letters in the free, bound or total TPC indicate significant differences (*p* < 0.05).

The pulps of the six melon varieties differed significantly (*p* < 0.05) in terms of free, bound, and total TPC (Figure 1). The free TPC was in the range of 15.58–57.74 mg GAE/100 g DW. Naixiangmi and Yugu present the highest free TPC followed by Hongguan, Meilong, and Xiaomi No. 25. Xizhoumi No. 25 had the lowest free TPC, which was significantly less than the other varieties (*p* < 0.05). This huge discrepancy in free TPC among the six melon varieties may be ascribed to variations in their genes regulating phenolic compound biosynthesis [13]. Prior studies reported significant discrepancies in free TPC exhibited among melon varieties [3,7,8,9]. The free TPC of the pulps of melons purchased in Malaysia and the UK was 14.19 mg GAE/100 g DW and 10.19 mg GAE/100 g Fresh Weight (FW), respectively [14,15]. These figures were lower than the ones obtained in the current study. However, another study reported a free TPC of 313.45 mg GAE/100 g DW for Brazilian melon [10], a value substantially larger than those obtained in the present study. The foregoing discrepancies may be attributed to relative differences among melon cultivars in terms of the expression levels of the genes regulating phenolic compound biosynthesis, harvest location and period, and free phenolic compound extraction method [13].

The bound TPC significantly varied in the range of 25.78–289.52 mg GAE/100 g DW. Meilong had the highest bound TPC and it was 10.15 times higher than that of Xizhoumi No. 25. Hongguan had the second highest bound TPC and it was 0.89-fold that of Meilong. The bound TPC of Naixiangmi and Xiaomi No. 25 were about 0.20-fold that of Meilong, though no significant differences were observed. (*p* > 0.05). The total TPC of all six melon varieties was in the range of 41.36–315.96 mg GAE/100 g DW. Of these, Meilong and Xizhoumi No. 25 had the highest and lowest, respectively, and the latter was 15.34% of the former. Significant differences existed in the bound TPC in the pulps of three melon cultivars in Japan [9]. Similar bound TPC trends were reported for different varieties of other fruits, such as litchi [16].

Plant phenolic compounds usually occur in free and bound forms. Significant differences in the proportion of bound TPC in total TPC were reported for different types of fruits and various cultivars of the same fruit [16,17]. An earlier study reported that the phenolic compounds in ten common fruits were mainly in free form and, to a far lesser extent, in bound form. The proportions of free TPC in total TPC were in the ranges of 3.81–38.10% for melon [17] and 8.60–36.9% for 13 litchi varieties [16]. The free phenolic form was the major contributor to the total TPC in three melon cultivars planted in Japan [9]. The percentage of bound TPC in total TPC decreased with the advancing melon growth stage [9]. However, the present study showed that this proportion was significantly different among the six melon varieties examined (*p* < 0.05). Meilong and Hongguan had the highest proportions of bound TPC in total TPC (91.61 ± 1.75% and 89.38 ± 0.33%, respectively), followed by Yugu (78.78 ± 2.24%), Xizhoumi (62.31 ± 0.85), Xiaomi (57.26 ± 3.80%), and Naixiangmi (37.71 ± 0.73) (*p* < 0.05). In Kainth fruit pulp, pineapple, blackberry, black raspberry, and blueberry, bound phenolics represent a major contributor to the TPC [17,18,19]. Red sorghum varieties widely differed in the proportions of bound TPC in their total TPC. This discovery was consistent with the findings of Meilong and Naixiangmi [20].

### 3.2. Phenolic Compounds Identification

Thirty phenolic compounds were identified in the present study (Table 1, Figures S3 and S4). Of these, 6 phenolic acids, 11 flavonoids, and 1 stilbene existed in free form while 12 phenolic acids, 15 flavonoids, and 1 stilbene were in bound form. Another four unknown compounds were also detected. To our best knowledge, bound phenolic compounds in melon pulp were identified for the first time in this study.

Six benzoic acids and their derivatives were characterized in the bound phenolic compound fraction. Of these, ethyl vanillin, vanillic acid, and syringic acid were also detected in the free phenolic compound fraction. The precursor and the product ion of peak 1 were observed at *m*/*z* 136.89 and 108.04, respectively, which reflected the loss of an aldehyde group. The peak was deduced to be protocatechualdehyde [21]. Peaks 2, 4, and 6 at *m*/*z* 153.10, 169.00, and 199.10 and their respective product ions at *m*/*z* 109.30, 125.00, and 155.10 corresponding to CO_2_ loss were preliminarily assigned as protocatechuic acid, vanillic acid, and syringic acid, respectively [22]. Ethyl vanillin (Peak 3) was distinguished by the −29 Th loss corresponding to the ethyl group (*m*/*z* 136.21) and the subsequent CO_2_ loss (*m*/*z* 92.05) [23]. Peak 5 corresponds to a precursor ion at *m*/*z* 153.00 and its product ion at *m*/*z* 125.00 reflecting CO loss, so it was tentatively named vanillin [24]. Seven hydroxycinnamic acids and their derivatives were characterized. All of these except cynarin were identified in the bound phenolic compound fraction, whereas cynarin, *p*-coumaric acid, and chlorogenic acid were detected in the free phenolic compound fraction. Peaks 7−11 were tentatively identified as *trans*-cinnamic, *p*-coumaric, ferulic, sinapic, and caffeic acids, respectively, on the basis of their deprotonated ions [M−H]^−^ at *m*/*z* 146.95, 163.10, 193.00, 223.00, and 179.10 and their product ions at *m*/*z* 118.90 [M−H−CO]^−^ (peak 7), 119.00 [M−H−CO_2_]^−^, 91.00 [M−H−CO_2_−C_2_H_4_]^−^ (peak 8), 177.90 [M−H−CH_3_]^−^, 149.00 [M−H−CO_2_]^−^ (peak 9), 193.00 [M−H−CH_3_−CH_3_]^−^, 149.00 [M−H−CH_3_−CH_3_−CO_2_]^−^ (peak 10), and 135.00 [M−H−CO_2_]^−^ (peak 11) [22,25,26]. Cynarin (peak 12) is the product of the esterification of one quinic acid and two caffeic acid molecules and was characterized by its parent ion at *m*/*z* 515.30, a quinic acid fragment (*m*/*z* 191.01), and a caffeoylquinic acid fragment (*m*/*z* 353.00) [27]. Chlorogenic acid (peak 13) comprises quinic acid and caffeic acid linked by an ester bond and was characterized by its parent ion (*m*/*z* 353.20), a quinic acid fragment (*m*/*z* 191.00), and a caffeic acid fragment (*m*/*z* 179.20) [26].

Epicatechin was tentatively identified from the CO_2_ loss (*m*/*z* 244.90) and retro Diels-Alder (RDA) fragmentation ([M−H−152]^−^) [28]. Peaks 15 and 16 correspond to the identical precursor ions at *m*/*z* 305.10 and the same fragments at *m*/*z* 179.00 [M−H−C_6_H_6_O_3_]^−^ and 124.98 [M−H−152]^−^, depending on the different retention times, the peaks represented (-)-epigallocatechin and (-)-gallocatechin, respectively [28]. Epigallocatechin gallate (peak 17) was distinguished by its parent ion [M−H]^−^ at *m*/*z* 457.00 and its product fragments at *m*/*z* 331.00 [M−H−C_6_H_6_O_3_]^−^, *m*/*z* 169.00 [gallic acid−H]^−^, and *m*/*z* 125.00 [gallic acid−H−CO_2_]^−^ [28]. A fragment ion observed at *m*/*z* 288.99 on account of the loss of (epi)catechin, was characterized as procyanidin [29]. Myricetin (peak 19) was featured by its precursor ion [M−H]^−^ at *m*/*z* 317.00 and its product fragments at *m*/*z* 179.00 and 151.00 resulting from typical RDA fragmentation [26]. Peaks 20, 21, and 24 were designated kaempferol-3-O-rutinoside, isorhamnetin-3-O-glucoside, and hesperidin, respectively, based on glycoside loss from the aglycone. This finding was coherent with the MS data reported by Olate-Gallegos et al. [29]. Taxifolin (peak 22) was marked by its parent ion [M−H]^−^ at *m*/*z* 302.83 and its fragments at *m*/*z* 285.00 [M−H−H_2_O]^−^, 176.90 [M−H−C_6_H_6_O_3_]^−^, and 124.98 [C_6_H_6_O_3_−H]^−^. These were previously reported by Olate-Gallegos et al. [29]. Taxifolin-7-O-rhamnoside (peak 23) was identified from the taxifolin fragment (*m*/*z* 303.00) resulting from rhamnoside (−146 Th) loss, H_2_O loss from the taxifolin fragment (*m*/*z* 284.90), and [C_6_H_6_O_3_−H]^−^ ion (*m*/*z* 124.98) [29]. Peak 25 was distinguished by its precursor ion [M+H]^+^ at *m*/*z* 271.00 and its product fragment at *m*/*z* 122.90 and was tentatively designated baicalein, which was in accordance with previously reported MS data [29]. Peak 26 was characterized as hinokiflavone by the precursor ion [M−H]^−^ at *m*/*z* 537.00 and fragment ion at *m*/*z* 284.00, consistent with the MS data published by a previous study [12]. Cosemetin (apigenin-7-O-glucoside) (peak 27) was distinguished by glucoside loss (−162 Th) from the aglycone apigenin [29]. The anthocyanidins (peaks 28 and 29) were determined on the basis of their parent ions [M+H]^+^ at *m*/*z* 286.90, 306.92 and their product ions with the corresponding fragments [M+H−150]^+^ (*m*/*z* 137.00) and [M+H−150−CO]^+^ (*m*/*z* 109.10) for cyanidin [30] and [M+H−152]^+^ (*m*/*z* 155.07) and [M+H−152−CO]^+^ (*m*/*z* 126.99) for leucocyanidin [31]. *Trans*-resveratrol (peak 30) displayed a protonated precursor [M+H]^+^ at *m*/*z* 229.00 and product fragments at *m*/*z* 135.00 and 107.10, resulting from phenol loss and hydroxytropylium ion formation [22].

Of the 18 free phenolic compounds detected herein, 12 were detected in the melon pulp in the previous study. Free chlorogenic acid, vanillic acid, syringic acid, *p*-coumaric acid, epicatechin, and resveratrol were identified by HPLC of the pulps of melons from Tunisia, Turkey, and the USA [8,32,33,34]. Nineteen free phenolic compounds including protocatechuic acid, apigenin-7-O-glucoside, and *p*-coumaric acid were assigned by HPLC from the pulp of a Maazoun melon cultivar planted in Tunisia [33,34]. HPLC-MS revealed free hesperidin and isorhamnetin-3-O-glucoside in the pulps of melon cultivars planted in Spanish and the USA, respectively [35,36]. Free aempferol-3-O-rutinoside and myricetin were detected in the pulp of melon from Egypt. Free quercitrin, isorhamnetin-3-O-glucoside, and apigenin-7-O-glucoside were found in melon peel [2]. To our best knowledge, the present study first detected cyanidin, ethyl vanillin, gallocatechin, epigallocatechin gallate, taxifolin, and hinokiflavone in the melon pulp. HPLC-MS did not disclose cynarin (1,3-O-dicaffeoylquinic acid) in the melon pulp. However, caffeic acid derivatives, such as caffeic acid-O-hexoside, in melon peel and esterified products of hydroxycinnamic and quinic acids, such as feruloylquinic acid and coumaroylquinic acid, were previously detected in melon pulp [2]. Gallocatechin, epigallocatechin gallate, taxifolin, and hinokiflavone were found in various fruits [37]. Amentoflavone is a hinokiflavone isomer that occurs in free form in melon pulp and peel [34,38].

To our best knowledge, there was no previously published research on the profile of bound phenolic compounds in the melon pulp. Hence, the present study was one of the first to provide a report on the identification of 28 individual phenolic compounds in melon pulp by UHPLC-QQQ-MS. Free protocatechuic acid, chlorogenic acid, vanillic acid, syringic acid, *p*-coumaric acid, caffeic acid, *trans*-cinnamic acid, ferulic acid, sinapic acid, resveratrol, apigenin-7-O-glucoside, hesperidin, isorhamnetin-3-O-glucoside, kaempferol-3-O-rutinoside, and myricetin were detected in melon pulp extracts [2,8,34,35]. Protocatechualdehyde, taxifolin, hinokiflavone, baicalein, and the flavan-3-ols gallocatechin, epigallocatechin, epigallocatechin gallate, and procyanidin B1 were detected in various fruits [37]. Here, bound vanillin, ethyl vanillin, taxifolin-7-O-rhamnoside, cyanidin, and leucocyanidin were found in melon pulp for the first time. Free vanillin and ethyl vanillin were observed in potato and cocoa powder, respectively [39,40]. Here, taxifolin-7-O-rhamnoside aglycone was identified in the bound phenolic compound fraction of melon pulp. Cyanidin was observed in cranberry beans [30].

### 3.3. Quantification of Individual Phenolic Compounds

Table 2 shows considerable differences in the number and concentration of individual free and bound phenolic compounds in the pulps of six melon varieties. We detected 16–23 types of individual phenolic compounds embracing phenolic acids, flavonoids, and stilbene. Of these, 7–10 compounds were in free form and 7–14 compounds were in bound form. Meilong and Xizhoumi No. 25 contained ≤ 23 individual phenolic compounds followed by Hongguan and Yugu (19), Naixiangmi (17), and Xiaomi No. 25 (16). HPLC or HPLC-MS disclosed 6–19 free phenolic compounds in melon pulp and peel [2,8,32,33,34,38]. In contrast, no information regarding bound phenolic compounds in melon has been reported.

Xizhoumi No. 25 contained the most diverse phenolic compounds in the free form while Hongguan and Yugu contained the least (7). Cosemetin, hinokiflavone, and chlorogenic acid were the paramount free phenolic compounds detected in all six melon varieties. Cosemetin was detected in all melon cultivars except Xizhoumi No. 25 and Xiaomi No. 25. and it accounted for 93.98% and 67.50% of the free phenolic compound content in Hongguan and Yugu, respectively. These proportions were significantly (*p* < 0.05) larger than those for Naixiangmi and Meilong. The hinokiflavone content was in the range of 66.64–152.60 μg/g DW, in the order Yugu > Xizhoumi No. 25 > Naixiangmi > Meilong > Xiaomi No. 25 > Hongguan, and comprised 3.64–58.32% of the free phenolic compound content. Hinokiflavone was the principal free phenolic compound in Naixiangmi (58.32%). Chlorogenic acid is ubiquitous in fruits, vegetables, and coffee, and also was detected in all six varieties of melon, constituting 0.13–44.54% of the free phenolic compound content. Yugu had significantly (*p* < 0.05) higher ethyl vanillin content (123.90 μg/g DW) than Xiaomi No. 25 (53.27 μg/g DW), Xizhoumi No. 25 (46.48 μg/g DW), or Naixiangmi (19.60 μg/g DW). Ethyl vanillin was also the major phenolic acid contributor in Yugu (≤99.08%). Chlorogenic acid and cosemetin accounted for 16.11–54.21% and 0.52–6.04% of the free phenolic compound content in melon pulp [33]. Amentoflavone (hinokiflavone isomer), cosemetin (293.40 μg/g DW), chlorogenic acid (82.50 μg/g DW), and gallic acid accounted for 27.90%, 2.42%, 1.75%, and 23.43% of the free phenolic compound content, respectively, in melon pulp [34]. Gallic acid, catechin, and rutin represented the major phenolic constituents, with the sum of their quantities comprising ~75% of the free phenolic compound content [32]. Benzoic acid (54.58%), vanillic acid (13.14%), epicatechin (6.74%), ferulic acid (6.70%), and *p*-coumaric acid (5.88%) constituted the prevailing free phenolic compounds in standard melon. Rutin (28.27–31.30%), benzoic acid (13.65–23.82%), vanillic acid (15.64–20.59%), ferulic acid (7.65–9.15%), and *p*-coumaric acid (8.07–10.23%) were the major free phenolic compounds in hybrid as well as a grafted melon. Hence, the free phenolic compounds and compositions significantly differ among melon varieties [8].

Most of the free phenolic compounds in melon pulp are substituted phenolic acids and flavonoids. Nevertheless, there is no consensus on the major phenolic class in this fruit tissue [8,32,33,34]. It was reported that the phenolic acid content was substantially higher than the flavonoid content in melon pulp [8,33]. Another report showed the opposite result [32]. Recent work indicated that the phenolic acid content (275.70 μg/g DW) and flavonoid content (243.40 μg/g DW) were nearly equal in melon pulp [34]. In the current research, the ratios of free phenolic acid content to free phenolic compound content were in the range of 51.08–60.97% for Xizhoumi No. 25 and Xiaomi No. 25, and in the range of 1.99–37.03% for the other four varieties. The proportion of the number of phenolic acids to the number of phenolic compounds in free form ranged from 22.22% to 50.00%. Thus, flavonoids were the predominant free phenolic compounds in most melon varieties. All six melon varieties were cultivated under similar environmental conditions in Hainan Province and harvested at maturity during the same period. Therefore, the observed differences in their phenolic profiles may be attributed to genetic factors. The discrepancies in the free phenolic profiles noted in this study and past studies could be attributed to genetic factors, planting location and period, extraction solvent type, and assisted extraction technology used [13].

As shown in Table 2, Meilong contained the most bound phenolic compounds while Xiaomi No. 25 had the fewest (7). Hinokiflavone was the predominant bound phenolic compound. It was detected in all six varieties in the range of 67.83–238.70 μg/g DW and accounted for 63.49–92.89% of the bound phenolic compound content. Yugu had the highest bound hinokiflavone content and it was 3.52-fold higher than that of Naixiangmi (*p* < 0.05). Meilong, Hongguan, and Yugu contained 47.81, 19.53, and 16.24 μg/g DW bound caffeic acid, respectively, which accounted for 16.61%, 9.86%, and 5.36% of their bound phenolic compound content, respectively. For all six varieties, the ratios of bound phenolic acid content to bound phenolic compound content varied from 1.83 to 28.98%.

The ranges of the content of free and bound phenolic compounds were 193.20–1830.11 μg/g DW and 87.86–303.74 μg/g DW, respectively. The proportions of them in total content of (free and bound) phenolic compounds were in the ranges of 41.38–90.23% and 9.77–58.62%, respectively. Hongguan exhibited the maximum content of free phenolic compound and total content of phenolic compounds, while Yugu possessed the maximum content of bound phenolic compound (*p* < 0.05). Xiaomi No. 25, Naixiangmi, and Naixiangmi showed the lowest free and bound phenolic compound content, and total content of phenolic compounds, respectively (*p* < 0.05). The content of the free phenolic compound was about 71% of the content of the bound phenolic compound in Meilong. However, for the other varieties, the free phenolic compound content was 1.60–9.24 times as high as the bound phenolic compound content. By contrast, the proportions of bound phenolic compound content in the total content of phenolic compounds were in the range of 37.71–91.61%. Hence, there were high concentrations of certain unidentified bound phenolic compounds.

### 3.4. Antioxidant Activity

**Figure 2:**
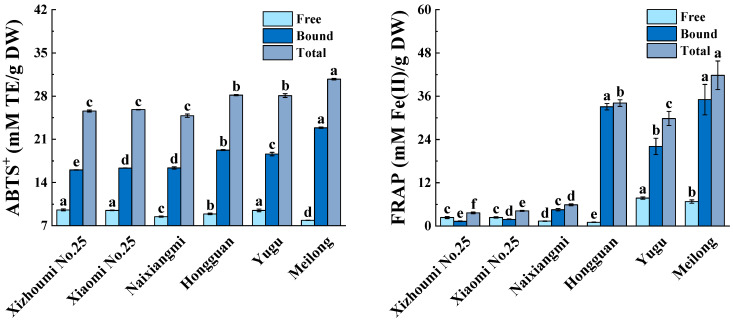
Comparison of free, bound, and total antioxidant activity of six melon varieties. Different letters in free, bound, or total antioxidant activity indicate significant differences (*p* < 0.05).

The significant free, bound, and total FRAP and ABTS^+^ values (*p* < 0.05) for the six melon varieties are plotted in Figure 2. The free, bound, and total FRAP were in the ranges of 1.01–7.74, 1.29–35.06, and 3.62–41.82 mM Fe(II)E/g DW, respectively. Yugu was found to contain the largest free FRAP value, while Hongguan and Meilong had the highest bound FRAP values. Meilong had the highest total FRAP value, followed by Hongguan, Yugu, Naixiangmi, Xiaomi No. 25, and Xizhoumi No. 25 (*p* < 0.05). The free, bound, and total ABTS^+^ values were within the ranges of 7.88–9.55, 16.04–22.88, and 24.83–30.76 mM TE/g DW, respectively. Meilong possessed the maximum bound and total ABTS^+^ values, whereas Yugu, Xiaomi No. 25, and Xizhoumi No. 25 had the lowest free ABTS^+^ values. Significant discrepancies were revealed in the antioxidant capacities of the free and bound extracts of various melon cultivars [3,7,8,9]. The percentage contributions of the bound antioxidant ability to the total antioxidant ability were 35.61–97.3% (FRAP) and 62.68–74.38% (ABTS^+^). Hongguan had the highest FRAP value, followed by Meilong. The latter had the highest ABTS^+^ value. These results were closely associated with the proportion of bound TPC in the total TPC. The percentage contributions of the bound antioxidant ability to the total antioxidant ability were in the range of 0.80–1.50% for three melon varieties planted in Japan, with the proportion of bound TPC in the total TPC of 6.25–10.45% [9]. The percentages of bound to total antioxidant capacity varied significantly among different varieties of litchi, blackberry, black raspberry, and blueberry as well [16,18]. Bound phenolic compounds accounted for most of the antioxidant capacity of blackberry, black raspberry, and blueberry [18]. In contrast, free phenolic compounds were the primary contributors to the antioxidant ability of litchi [16]. The present study was one of the first to detect the potent antioxidant activity of the bound phenolic extracts of melon pulp. This discovery suggests that melon has health-promoting efficacy [4].

### 3.5. Correlations among Free, Bound, Total TPC and Antioxidant Activity

**Figure 3:**
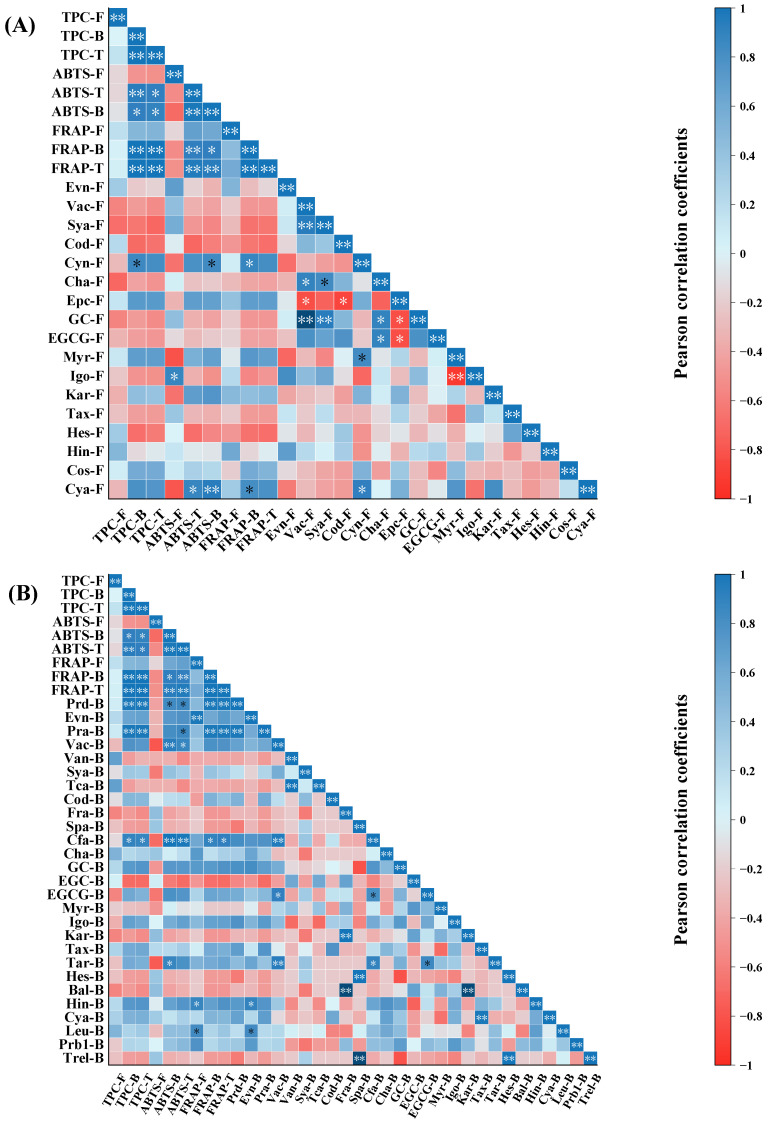
Correlation plot among (**A**) the content of free individual phenolic compounds, TPC, and antioxidant activity; and (**B**) the content of bound individual compounds, TPC, and antioxidant activity. * and ** indicate significance at *p* < 0.05 and *p* < 0.01, respectively. F: free, B: bound, T: total (free plus bound). Note: F: free, B: bound, T: total (free plus bound), Prd-Protocatechualdehyde, Pra-protocatechuic acid, Evn-ethyl vanillin, Vac-vanillic acid, Van-vanillin, Sya-syringic acid, Tca-trans-cinnamic acid, Cod-p-coumaric acid, Fra-ferulic acid, Spa-sinapic acid, Cfa-caffeic acid, Cha-chlorogenic acid, GC-gallocatechin, EGC-epigallocatechin, EGCG-epigallocatechin gallate, Myr-myricetin, Kar-kaempferol-3-O-rutinoside, Tax-taxifolin, Tar-taxifolin 7-O-rhamnoside, Hes-hesperidin, Bal-baicalein, Hin-hinokiflavone, Cya-cyanidin, Leu-leucocyanidin, Prb1-procyanidin B1, Trel-trans-resveratrol.

Pearson’s correlation coefficients were determined for free, bound, total TPC, and antioxidant activity (Figure 3). Significant positive correlations were presented among total TPC and the bound FRAP (*p* < 0.01), total FRAP (*p* < 0.01), bound ABTS^+^ (*p* < 0.05), and total ABTS^+^ (*p* < 0.05). Bound TPC showed a positive correlation with bound and total FRAP (*p* < 0.01; *p* < 0.01) and ABTS^+^ significantly (*p* < 0.05; *p* < 0.01). However, antioxidant capacity was not correlated with free TPC. The preceding results indicated that bound phenolic compounds substantially contributed to the total antioxidant activity of melon pulp. Positive correlations among antioxidant activity and free, bound, and total TPC were previously reported [9]. On the other hand, another study reported only a slight positive correlation between antioxidant activity and free TPC [41].

### 3.6. Correlations among Individual Phenolic Compound Content, TPC, and Antioxidant Activity

Figure 3A,B show the correlations among free and bound individual phenolic compound content, TPC, and antioxidant capacity. Except for those between isorhamnetin-3-O-glucoside and free ABTS^+^ value and between cyanidin and total ABTS^+^ value, the correlations between free individual phenolic compound content and antioxidant activity were weak. Overall, the correlations were weak between free individual phenolic compound content and antioxidant capacity. Bound protocatechualdehyde, protocatechuic acid, and caffeic acid had a positive correlation with bound TPC and total TPC. Protocatechualdehyde and caffeic acid were also correlated with bound and total ABTS^+^, and bound and total FRAP values positively. Protocatechuic acid was found to be positively correlated with total ABTS^+^ values and bound and total FRAP values. Vanillic acid was shown to have a positive correlation with bound and total ABTS^+^ values. Ttaxifolin-7-O-rhamnoside was shown to have a positive correlation with bound ABTS^+^ values. Hence, bound protocatechualdehyde, protocatechuic acid, caffeic acid, vanillic acid, and taxifolin-7-O-rhamnoside might substantially contribute to the antioxidant capacity of melon pulp.

### 3.7. Principal Component Analysis and Hierarchical Cluster Analyses

**Figure 4:**
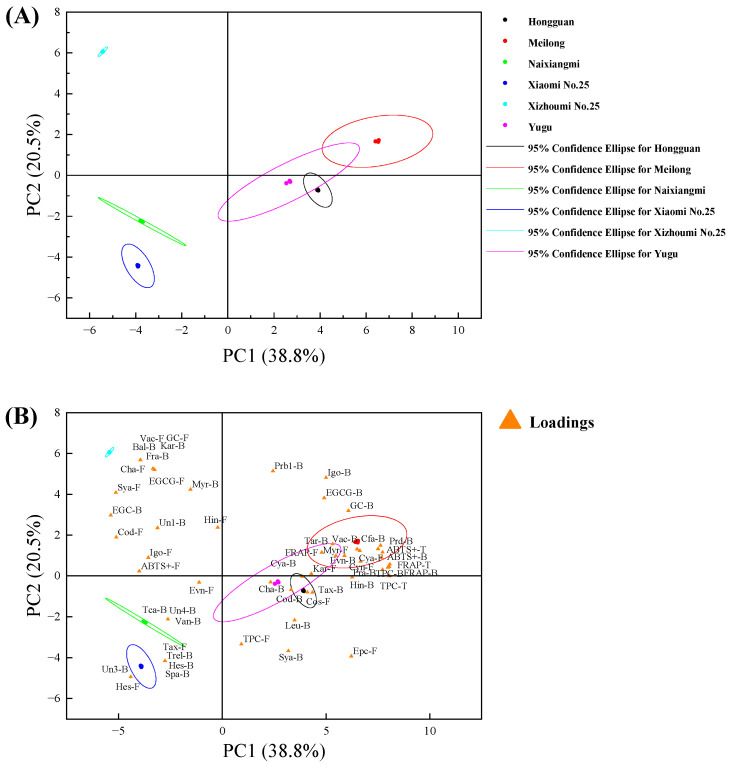
Principal component analysis (**A**) Scores plot showing the sample clustering; (**B**) Loadings plot reflecting the influence of a particular parameter. F: free, B: bound, T: total (free plus bound), Prd-Protocatechualdehyde, Pra-protocatechuic acid, Evn-ethyl vanillin, Vac-vanillic acid, Van-vanillin, Sya-syringic acid, Tca-trans-cinnamic acid, Cod-p-coumaric acid, Fra-ferulic acid, Spa-sinapic acid, Cfa-caffeic acid, Cyn-cynarin, Cha-chlorogenic acid, Epc-epicatechin, GC-gallocatechin, EGC-epigallocatechin, EGCG-epigallocatechin gallate, Myr-myricetin, Kar-kaempferol-3-O-rutinoside, Igo-isorhamnetin-3-O-glucoside, Tax-taxifolin, Tar-taxifolin 7-O-rhamnoside, Hes-hesperidin, Bal-baicalein, Hin-hinokiflavone, Cos-cosemetin, Cya-cyanidin, Leu-leucocyanidin, Prb1-procyanidin B1, Trel-trans-resveratrol.

Principal component analyses (PCA) and hierarchical cluster analyses (HCA) have been extensively utilized to identify the similarities and differences among fruit varieties based on their TPC and antioxidant ability [32,37]. Here, the free, bound, and total TPC, the content of individual phenolic compounds, and the antioxidant activity (FRAP and ABTS^+^) of the six melon varieties planted in Hainan Province comprised the initial 6 × 56 matrix. Three principal components (PCs) were generated, and PC1 and PC2 explained 38.80% and 20.50% each of the overall variances, respectively (Figure 4). Figure 4A depicts the score plot indicating sample clustering, while Figure S2 shows the similarity and clustering of the six melon varieties. Figure 4B shows a loading plot that reflects the influence of each parameter on the PC and the samples during clustering. Xizhoumi No. 25 is in the upper left area of Figure 4A and was distinguished by the fact that it contained higher free syringic acid, chlorogenic acid, and epigallocatechin gallate content than the other varieties. Furthermore, only Xizhoumi No. 25 contained free vanillic acid, gallocatechin, bound ferulic acid, and baicalein. The PCA and HCA disclosed that Meilong was also distinct from the other varieties. Xiaomi No. 25 and Naixiangmi had similar phenolic compound composition and content (including free hesperidin) but differed in terms of their free ethyl vanillin and taxifolin, and bound vanillin content. Moreover, the PCA and HCA revealed similarities between Yugu and Hongguan. Figure S2 shows that the six melon varieties were divided into four taxa represented by different colored lines. These melon cultivars differed in terms of the metabolic pathways of their phenolic compounds [27].

## 4. Conclusions

Significant variations are revealed in the free, bound, and total TPC as well as the antioxidant capacity of the six melon varieties. The bound TPC was significantly greater than that of free TPC in all melon cultivars tested except for Naixiangmi. There were strong positive correlations among bound and total TPC and antioxidant activity. Hence, melon pulp has potential health-promoting efficacy. UHPLC-QQQ-MS disclosed 16–23 individual phenolic compounds. Of these, 7–10 were in free form while another 7–14 were in bound form. Cosemetin, hinokiflavone, and chlorogenic acid were the principal free phenolic compounds, while hinokiflavone was the predominant bound phenolic compound. PCA and HCA divided the six melon varieties into four taxa. Meilong exhibited the maximum bound TPC, total TPC, bound phenolic compound content and the strongest bound and total antioxidant activity, while Hongguan had the second highest bound TPC, total TPC, bound phenolic compound content, and the highest free phenolic compound content and total content of phenolic compounds. Therefore, these melon cultivars demonstrated promise as food sources with health-promoting efficacy. It is believed that the findings of the present study will contribute scientific information on the nutritional and medicinal value of melon cultivated in Hainan Province.

## Figures and Tables

**Table 1:** Identification of individual phenolic compounds in the pulp of six melon varieties by UHPLC-QQQ-MS.

Phenolic Compounds Classes	Peak No.	Tentative Compounds	λ_max_ (nm)	ESI Mode	Parent Ion	Fragment Ion	Reference	Form
benzoic acid and derivative	1	protocatechualdehyde	274, 308	−	136.89	108.04	[21], standard	B
	2	protocatechuic acid	260, 295	−	153.10	109.30	[22], standard	B
	3	ethyl vanillin	280, 310	−	165.00	136.21, 92.05	[23], standard	F, B
	4	vanillic acid	259, 291	+	169.00	125.00	[22], standard	F, B
	5	vanillin	275	+	153.00	125.00	[24], standard	B
	6	syringic acid	275	+	199.10	155.10	[22], standard	F, B
hydroxycinnamic acid and derivative	7	*trans*-cinnamic acid	278, 306	−	146.95	118.90, 77.00, 40.10	[25], standard	B
	8	*p*-coumaric acid	270, 307	−	163.10	119.00, 91.00	[26], standard	F, B
	9	ferulic acid	299, 323	−	193.00	177.90, 149.00,	[26], standard	B
	10	sinapic acid	244, 323	−	223.00	193.00, 149.00, 121.00	[26], standard	B
	11	caffeic acid	299, 323	−	179.10	135.00, 79.00	[26], standard	B
	12	cynarin	242, 304	−	515.30	353.00, 191.01	[27], standard	F
	13	chlorogenic acid	241, 322	−	353.20	191.00, 182.90, 179.20	[26], standard	F, B
flavan-3-ol	14	epicatechin	278	−	289.10	244.90, 137.10	[28], standard	F
	15	gallocatechin	270	−	305.10	179.00, 124.98	[28], standard	F, B
	16	epigallocatechin	270	−	305.10	179.00, 124.98	[28], standard	B
	17	epigallocatechin gallate	274	−	457.00	331.00, 169.00, 125.00	[28], standard	F, B
	18	procyanidin B1	280	−	577.30	407.10, 288.99	[29], standard	B
flavonol	19	myricetin	251, 375	−	317.00	179.00, 151.00, 136.90	[26], standard	F, B
	20	kaempferol-3-O-rutinoside	266, 348	−	592.91	284.80, 254.91	[29], standard	F, B
	21	isorhamnetin-3-O-glucoside	254, 343	−	477.00	313.90, 284.90, 242.80	[29], standard	F, B
dihydroflavonol	22	taxifolin	288	−	302.83	285.00, 176.90, 124.98	[29], standard	F, B
	23	taxifolin-7-O-rhamnoside	286	−	449.00	303.00, 284.90, 124.98	[29], standard	B
flavanone	24	hesperidin	283, 327	−	609.30	301.10	[26], standard	F, B
flavone	25	baicalein	276	+	271.00	122.90	[29], standard	B
	26	hinokiflavone	272, 330	−	537.00	417.00, 284.00	[12], standard	F, B
	27	cosemetin	270, 334	−	430.90	268.90	[29], standard	F, B
anthocyanidin	28	cyanidin	276, 530	+	286.90	137.00, 109.10	[30], standard	F, B
	29	leucocyanidin	280	+	306.92	126.99, 155.07	[31], standard	B
stilbene	30	*trans*-resveratrol	280, 306	+	229.00	135.00, 107.10	[22], standard	F, B
	31	unknown1	256	−	1083.50	1083.50		F, B
	32	unknown2	257, 356	−	463.00	270.99		F
	33	unknown3	286	−	717.50	321.00		B
	34	unknown4	299,323	+	181.00	103.00		B

Note: F: free, B: bound.

**Table 2:** Contents of individual phenolic compounds in the pulp of six melon varieties determined by UHPLC-QQQ-MS.

Individual Phenolic Compounds	Form	Content (μg/g DW)					
		Xizhoumi No. 25	Xiaomi No. 25	Naixiangmi	Hongguan	Yugu	Meilong
protocatechualdehyde	B	2.70 ± 0.017 d	nd	1.99 ± 0.066 e	10.64 ± 0.076 a	8.54 ± 0.023 c	9.87 ± 0.038 b
protocatechuic acid	B	nd	nd	nd	5.94 ± 0.046 a	4.54 ± 0.15 c	4.80 ± 0.234 b
ethyl vanillin	F	46.48 ± 1.271 c	53.27 ± 2.171 b	19.60 ± 2.242 d	nd	123.90 ± 1.028 a	nd
	B	nd	nd	nd	nd	12.88 ± 0.782 a	12.88 ± 0.845 a
vanillic acid	F	0.38 ± 0.073 a	nd	nd	nd	nd	nd
	B	nd	nd	nd	2.24 ± 0.048 b	nd	5.90 ± 0.021 a
vanillin	B	nd	nd	0.20 ± 0.008 a	nd	nd	nd
syringic acid	F	2.94 ± 0.102 a	1.14 ± 0.11 b	nd	nd	nd	nd
	B	nd	1.94 ± 0.231 a	1.53 ± 0.295 b	2.26 ± 0.229 a	nd	2.16 ± 0.119 a
trans-cinnamic acid	B	nd	nd	3.97 ± 0.012 a	nd	nd	nd
p-coumaric acid	F	4.69 ± 0.035 b	nd	6.27 ± 0.077 a	nd	nd	nd
	B	nd	nd	nd	5.48 ± 0.031 a	nd	nd
ferulic acid	B	0.051 ± 0.002 a	nd	nd	nd	nd	nd
sinapic acid	B	nd	0.27 ± 0.001 a	nd	nd	nd	nd
caffeic acid	B	0.67 ± 0.016 d	nd	nd	19.53 ± 1.190 b	16.24 ± 1.228 c	47.81 ± 2.830 a
cynarin	F	nd	nd	nd	1.43 ± 0.021 a	nd	1.43 ± 0.015 a
chlorogenic acid	F	147.70 ± 6.900 a	44.28 ± 2.186 c	49.22 ± 2.233 c	34.94 ± 2.515 d	1.14 ± 0.047 e	70.00 ± 2.906 b
	B	nd	nd	nd	nd	1.33 ± 0.068 a	nd
the number of phenolic acids	F	5	3	3	2	2	2
	B	3	2	4	6	5	6
the content of phenolic acids	F	202.19 ± 8.208 a	98.69 ± 3.370 c	75.10 ± 3.100 d	36.37 ± 1.140 e	125.04 ± 1.700 b	71.43 ± 1.310 d
	B	3.42 ± 0.290 d	2.21 ± 0.178 d	7.69 ± 0.597 c	46.09 ± 1.860 b	43.53 ± 2.651 b	83.43 ± 3.217 a
epicatechin	F	nd	0.63 ± 0.015 b	0.27 ± 0.011 d	0.63 ± 0.014 b	0.58 ± 0.05 c	0.71 ± 0.016 a
gallocatechin	F	2.39 ± 0.042 a	nd	nd	nd	nd	nd
	B	2.62 ± 0.037 d	nd	2.49 ± 0.069 d	2.95 ± 0.077 c	4.64 ± 0.230 b	4.99 ± 0.098 a
epigallocatechin	B	2.62 ± 0.079 a	nd	2.49 ± 0.069 b	nd	nd	nd
epigallocatechin gallate	F	3.47 ± 0.189 a	nd	1.90 ± 0.120 b	nd	nd	1.55 ± 0.118 c
	B	1.56 ± 0.119 b	nd	nd	1.55 ± 0.100 b	nd	3.62 ± 0.198 a
procyanidin B1	B	1.32 ± 0.021 a	nd	nd	nd	1.35 ± 0.077 a	1.32 ± 0.048 a
myricetin	F	1.86 ± 0.120 c	nd	4.31 ± 0.180 b	6.04 ± 0.410 a	1.80 ± 0.097 c	5.87 ± 0.540 a
	B	4.98 ± 0.202 a	nd	4.31 ± 0.180 b	nd	nd	4.43 ± 0.110 a
kaempferol-3-O-rutinoside	F	nd	0.04 ± 0.001 b	nd	nd	nd	0.13 ± 0.002 a
	B	0.09 ± 0.001 a	nd	nd	nd	nd	nd
isorhamnetin-3-O-glucoside	F	2.01 ± 0.010 a	2.02 ± 0.015 a	nd	nd	2.03 ± 0.023 a	nd
	B	2.02 ± 0.160 a	0.24 ± 0.002 b	nd	2.04 ± 0.210 a	2.03 ± 0.200 a	2.04 ± 0.220 a
taxifolin	F	nd	0.72 ± 0.006 a	nd	nd	nd	nd
	B	nd	nd	nd	0.96 ± 0.016 a	0.71 ± 0.017 b	nd
taxifolin-7-O-rhamnoside	B	nd	nd	nd	nd	nd	0.28 ± 0.001 a
hesperidin	F	nd	2.91 ± 0.010 b	2.95 ± 0.012 a	nd	nd	nd
	B	nd	2.91 ± 0.009 a	nd	nd	nd	nd
baicalein	B	7.84 ± 0.027 a	nd	nd	nd	nd	nd
hinokiflavone	F	119.70 ± 2.446 b	77.00 ± 1.103 d	118.30 ± 0.150 b	66.64 ± 2.404 e	152.60 ± 4.620 a	104.30 ± 1.960 c
	B	84.00 ± 1.205 e	112.00 ± 1.740 d	67.83 ± 1.121 f	137.90 ± 2.146 c	238.70 ± 3.023 a	182.70 ± 2.754 b
cosemetin	F	nd	11.20 ± 1.187 c	nd	1719.90 ± 10.980 a	585.90 ± 11.540 b	18.06 ± 1.850 c
cyanidin	F	nd	nd	nd	0.54 ± 0.003 b	nd	1.18 ± 0.005 a
	B	1.12 ± 0.098 b	nd	nd	6.64 ± 0.657 a	6.87 ± 0.870 a	nd
leucocyanidin	B	nd	3.07 ± 0.400 b	3.07 ± 0.227 b	nd	5.92 ± 0.790 a	5.04 ± 0.920 a
the number of flavonoids	F	5	7	5	5	5	7
	B	10	4	5	6	7	8
the content of flavonoids	F	129.42 ± 3.100 c	94.52 ± 1.644 d	127.74 ± 2.415 c	1793.75 ± 13.500 a	742.91 ± 6.930 b	131.80 ± 4.757 c
	B	108.17 ± 2.530 e	118.21 ± 2.886 d	80.17 ± 1.847 f	152.03 ± 2.240 c	260.22 ± 3.980 a	204.43 ± 1.340 b
trans-resveratrol	B	nd	0.15 ± 0.001 a	nd	nd	nd	nd
the number of phenolic compounds	F	10	9	8	7	7	9
	B	13	7	9	12	12	14
	T	23	16	17	19	19	23
the content of phenolic compounds	F	331.61 ± 3.580 c	193.20 ± 1.824 e	202.83 ± 2.095 d	1830.11 ± 5.120 a	867.95 ± 5.855 b	203.23 ± 1.467 d
	B	111.59 ± 2.158 e	120.57 ± 2.006 d	87.86 ± 1.428 f	198.12 ± 2.753 c	303.74 ± 3.616 a	287.86 ± 2.536 b
	T	443.2 ± 1.254 d	313.77 ± 1.476 e	290.69 ± 1.897 f	2028.23 ± 13.357 a	1171.69 ± 8.755 b	491.09 ± 6.097 c

Note: nd: not detected, F: free, B: bound, T: total (free plus bound); different letters in the same row indicate significant differences (*p* < 0.05).

## Data Availability

Data are contained within the article.

## Supplementary Materials

The following supporting information can be downloaded at: https://www.mdpi.com/article/10.3390/foods12244446/s1, Figure S1: Photographic images of six melon varieties planted in the Hainan province of China; Figure S2: Hierarchical Cluster Analysis of free, bound, and total TPC, FRAP, ABTS+, and phenolic profiles of six melon varieties; Figure S3: Total ion chromatogram of free extracts of the pulp of six melon varieties analyzed by UHPLC-QQQ-MS in negative and positive modes; Figure S4: Total ion chromatogram of bound extracts of the pulp of six melon varieties analyzed by UHPLC-QQQ-MS in negative and positive modes.

## References

[1] FAO FAO Statistical Database. https://www.fao.org/faostat/zh/#data/QCL/visualize.

[2] Ezzat S.M., Raslan M., Salama M.M., Menze E.T., El Hawary S.S. (2019). In vivo anti-inflammatory activity and UPLC-MS/MS profiling of the peels and pulps of *Cucumis melo* var. *cantalupensis* and *Cucumis melo* var. *reticulatus*. J. Ethnopharmacol..

[3] Maietti A., Tedeschi P., Stagno C., Bordiga M., Travaglia F., Locatelli M., Arlorio M., Brandolini V. (2012). Analytical Traceability of Melon (*Cucumis Melo* Var *Reticulatus*): Proximate Composition, Bioactive Compounds, and Antioxidant Capacity in Relation to Cultivar, Plant Physiology State, and Seasonal Variability. J. Food Sci..

[4] Gomez-Garcia R., Campos D.A., Aguilar C.N., Madureira A.R., Pintado M. (2020). Valorization of melon fruit (*Cucumis melo* L.) by-products: Phytochemical and Biofunctional properties with Emphasis on Recent Trends and Advances. Trends Food Sci. Technol..

[5] Zhang B., Zhang Y., Li H., Deng Z., Tsao R. (2020). A review on insoluble-bound phenolics in plant-based food matrix and their contribution to human health with future perspectives. Trends Food Sci. Technol..

[6] Del Rio D., Rodriguez-Mateos A., Spencer J.P.E., Tognolini M., Borges G., Crozier A. (2013). Dietary (Poly)phenolics in Human Health: Structures, Bioavailability, and Evidence of Protective Effects Against Chronic Diseases. Antioxid. Redox Signal..

[7] Shivapriya M., Chidambara Murthy K.N., Vishnuvardana, Bhimanagouda S.P. (2021). Nutritional composition and health benefits of various botanical types of melon (*Cucumis melo* L.). Plants.

[8] Kolayli S., Kara M., Tezcan F., Erim F.B., Sahin H., Ulusoy E., Aliyazicioglu R. (2010). Comparative Study of Chemical and Biochemical Properties of Different Melon Cultivars: Standard, Hybrid, and Grafted Melons. J. Agric. Food Chem..

[9] Motomura Y., Sugawara J., Aikawa T., Nara K., Nishizawa T. Changes in free and bound phenolic contents and antioxidant activities of melon flesh dried at different stages of fruit growth. Proceedings of the 3rd Asia Pacific Symposium on Postharvest Research, Education and Extension (APS).

[10] Morais D.R., Rotta E.M., Sargi S.C., Schmidt E.M., Bonafe E.G., Eberlin M.N., Sawaya A.C.H.F., Visentainer J.V. (2015). Antioxidant activity, phenolics and UPLC-ESI(-)-MS of extracts from different tropical fruits parts and processed peels. Food Res. Int..

[11] Rolim P.M., Juca Seabra L.M.A., de Macedo G.R. (2020). Melon By-Products: Biopotential in Human Health and Food Processing. Food Rev. Int..

[12] Wu Y., Gao H., Wang Y., Peng Z., Guo Z., Ma Y., Zhang R., Zhang M., Wu Q., Xiao J. (2022). Effects of different extraction methods on contents, profiles, and antioxidant abilities of free and bound phenolics of *Sargassum polycystum* from the South China Sea. J. Food Sci..

[13] Yan Y., Pico J., Sun B., Pratap-Singh A., Gerbrandt E., Castellarin S.D. (2021). Phenolic profiles and their responses to pre- and post-harvest factors in small fruits: A review. Crit. Rev. Food Sci. Nutr..

[14] Shofian N.M., Hamid A.A., Osman A., Saari N., Anwar F., Dek M.S.P., Hairuddin M.R. (2011). Effect of Freeze-Drying on the Antioxidant Compounds and Antioxidant Activity of Selected Tropical Fruits. Int. J. Mol. Sci..

[15] Fundo J.F., Miller F.A., Garcia E., Santos J.R., Silva C.L.M., Brando T.R.S. (2018). Physicochemical characteristics, bioactive compounds and antioxidant activity in juice, pulp, peel and seeds of *Cantaloupe melon*. J. Food Meas. Charact..

[16] Zhang R., Zeng Q., Deng Y., Zhang M., Wei Z., Zhang Y., Tang X. (2013). Phenolic profiles and antioxidant activity of litchi pulp of different cultivars cultivated in Southern China. Food Chem..

[17] Sun J., Chu Y.F., Wu X., Liu R.H. (2002). Antioxidant and antiproliferative activities of common fruits. J. Agric. Food Chem..

[18] Ayoub M., de Camargo A.C., Shahidi F. (2016). Antioxidants and bioactivities of free, esterified and insoluble-bound phenolics from berry seed meals. Food Chem..

[19] Prakash O., Baskaran R., Kudachikar V.B. (2019). Characterization, quantification of free, esterified and bound phenolics in Kainth (*Pyrus pashia* Buch.-Ham. Ex D.Don) fruit pulp by UPLC-ESI-HRMS/MS and evaluation of their antioxidant activity. Food Chem..

[20] Li M., Xu T., Zheng W., Gao B., Zhu H., Xu R., Deng H., Wang B., Wu Y., Sun X. (2021). Triacylglycerols compositions, soluble and bound phenolics of red sorghums, and their radical scavenging and anti-inflammatory activities. Food Chem..

[21] Luo D., Mu T., Sun H. (2021). Profiling of phenolic acids and flavonoids in sweet potato (*Ipomoea batatas* L.) leaves and evaluation of their anti-oxidant and hypoglycemic activities. Food Biosci..

[22] Lambert M., Meudec E., Verbaere A., Mazerolles G., Wirth J., Masson G., Cheynier V., Sommerer N. (2015). A High-Throughput UHPLC-QqQ-MS Method for Polyphenol Profiling in Rose Wines. Molecules.

[23] Jager L.S.d., Perfetti G.A., Diachenko G.W. (2007). Determination of coumarin, vanillin, and ethyl vanillin in vanilla extract products: Liquid chromatography mass spectrometry method development and validation studies. J. Chromatogr. A.

[24] Flamini R., Vedova A.D., Cancian D., Panighel A., De Rosso M. (2007). GC/MS-positive ion chemical ionization and MS/MS study of volatile benzene compounds in five different woods used in barrel making. J. Mass Spectrom..

[25] Arivalagan M., Roy T.K., Yasmeen A.M., Pavithra K.C., Jwala P.N., Shivasankara K.S., Manikantan M.R., Hebbar K.B., Kanade S.R. (2018). Extraction of phenolic compounds with antioxidant potential from coconut (*Cocos nucifera* L.) testa and identification of phenolic acids and flavonoids using UPLC coupled with TQD-MS/MS. LWT Food Sci..

[26] Mattonai M., Parri E., Querci D., Degano I., Ribechini E. (2016). Development and validation of an HPLC-DAD and HPLC/ESI-MS2 method for the determination of polyphenols in monofloral honeys from Tuscany (Italy). Microchem. J..

[27] Ren M., Xu W., Zhang Y., Ni L., Lin Y., Zhang X., Huang M. (2020). Qualitative and quantitative analysis of phenolic compounds by UPLC-MS/MS and biological activities of *Pholidota chinensis* Lindl. J. Pharm. Biomed. Anal..

[28] Akhtar N., Thadhani V.M., Ul Haq F., Khan M.N., Ali S., Musharraf S.G. (2020). Rapid identification and quantification of bioactive metabolites in processed *Camellia sinensis* samples by UHPLC-ESI-MS/MS and evaluation of their antioxidant activity. J. Ind. Eng. Chem..

[29] Olate-Gallegos C., Barriga A., Vergara C., Fredes C., Garcia P., Gimenez B., Robert P. (2019). Identification of Polyphenols from Chilean Brown Seaweeds Extracts by LC-DAD-ESI-MS/MS. J. Aquat. Food Prod. Technol..

[30] Chen P.X., Tang Y., Marcone M.F., Pauls P.K., Zhang B., Liu R., Tsao R. (2015). Characterization of free, conjugated and bound phenolics and lipophilic antioxidants in regular- and non-darkening cranberry beans (*Phaseolus vulgaris* L.). Food Chem..

[31] Yang J., Qian D., Jiang S., Shang E.-x., Guo J., Duan J.-a. (2012). Identification of rutin deglycosylated metabolites produced by human intestinal bacteria using UPLC-Q-TOF/MS. J. Chromatogr. B-Anal. Technol. Biomed. Life Sci..

[32] Mohamed Ahmed I.A., Al Juhaimi F., Musa Ozcan M., Nurhan U., Elfadil E.B., Kashif G., Magdi A.O., Hesham A.A.S. (2021). A comparative study of bioactive compounds, antioxidant activity and phenolic compounds of melon (*Cucumis melo* L.) slices dehydrated by oven, microwave and infrared systems. J. Food Process. Preserv..

[33] Ganji S.M., Singh H., Friedman M. (2019). Phenolic Content and Antioxidant Activity of Extracts of 12 Melon (*Cucumis melo*) Peel Powders Prepared from Commercial Melons. J. Food Sci..

[34] Mallek-Ayadi S., Bahloul N., Baklouti S., Kechaou N. (2022). Bioactive compounds from *Cucumis melo* L. fruits as potential nutraceutical food ingredients and juice processing using membrane technology. Food Sci. Nutr..

[35] Ravindranath V., Singh J., Jayaprakasha G.K., Patil B.S. (2021). Optimization of Extraction Solvent and Fast Blue BB Assay for Comparative Analysis of Antioxidant Phenolics from *Cucumis melo* L.. Plants.

[36] Rodriguez-Perez C., Quirantes-Pine R., Fernandez-Gutierrez A., Segura-Carretero A. (2013). Comparative characterization of phenolic and other polar compounds in Spanish melon cultivars by using high-performance liquid chromatography coupled to electrospray ionization quadrupole-time of flight mass spectrometry. Food Res. Int..

[37] Serni E., Tomada S., Haas F., Robatscher P. (2022). Characterization of phenolic profile in dried grape skin of *Vitis vinifera* L. cv. Pinot Blanc with UHPLC-MS/MS and its development during ripening. J. Food Compos. Anal..

[38] Mallek-Ayadi S., Bahloul N., Kechaou N. (2017). Characterization, phenolic compounds and functional properties of *Cucumis melo* L. peels. Food Chem..

[39] Perez-Esteve E., Jesus Lerma-Garcia M., Fuentes A., Palomares C., Barat J.M. (2016). Control of undeclared flavoring of cocoa powders by the determination of vanillin and ethyl vanillin by HPLC. Food Control.

[40] Suo H., Peng Z., Guo Z., Wu C., Liu J., Wang L., Xiao J., Li X. (2022). Deep eutectic solvent-based ultrasonic-assisted extraction of phenolic compounds from different potato genotypes: Comparison of free and bound phenolic profiles and antioxidant activity. Food Chem..

[41] Selale H., Sigva H.O., Celik I., Doganlar S., Frary A. (2012). water-soluble antioxidant potential of melon lines grown in Turkey. Int. J. Food Prop..

