# Supplementary material for: Free and Bound Phenolic Profiles and Antioxidant Activities in Melon (Cucumis melo L.) Pulp: Comparative Study on Six Widely Consumed Varieties Planted in Hainan Province
Source: Foods. 2023 Dec 12;12(24):4446. doi: 10.3390/foods12244446 (PMC10742615; doi:10.3390/foods12244446)
Supplement: Supplementary file 1 [file foods-12-04446-s001.zip › foods-2749708-supplementary.pdf]

Supplementary material

"Free and bound phenolic profiles and antioxidant activities in melon (*Cucumis melo* L.) pulp: Comparative study on six widely consumed varieties planted in Hainan province"

Figure S1 Photographic images of six melon varieties planted in the Hainan province of China.

Figure S2 Hierarchical Cluster Analysis of free, bound, and total TPC, FRAP, $ABTS^+$, and phenolic profiles of six melon varieties.

Figure S3 Total ion chromatogram of free extracts of the pulp of six melon varieties analyzed by UHPLC-QQQ-MS in negative and positive modes.

Figure S4 Total ion chromatogram of bound extracts of the pulp of six melon varieties analyzed by UHPLC-QQQ-MS in negative and positive modes.

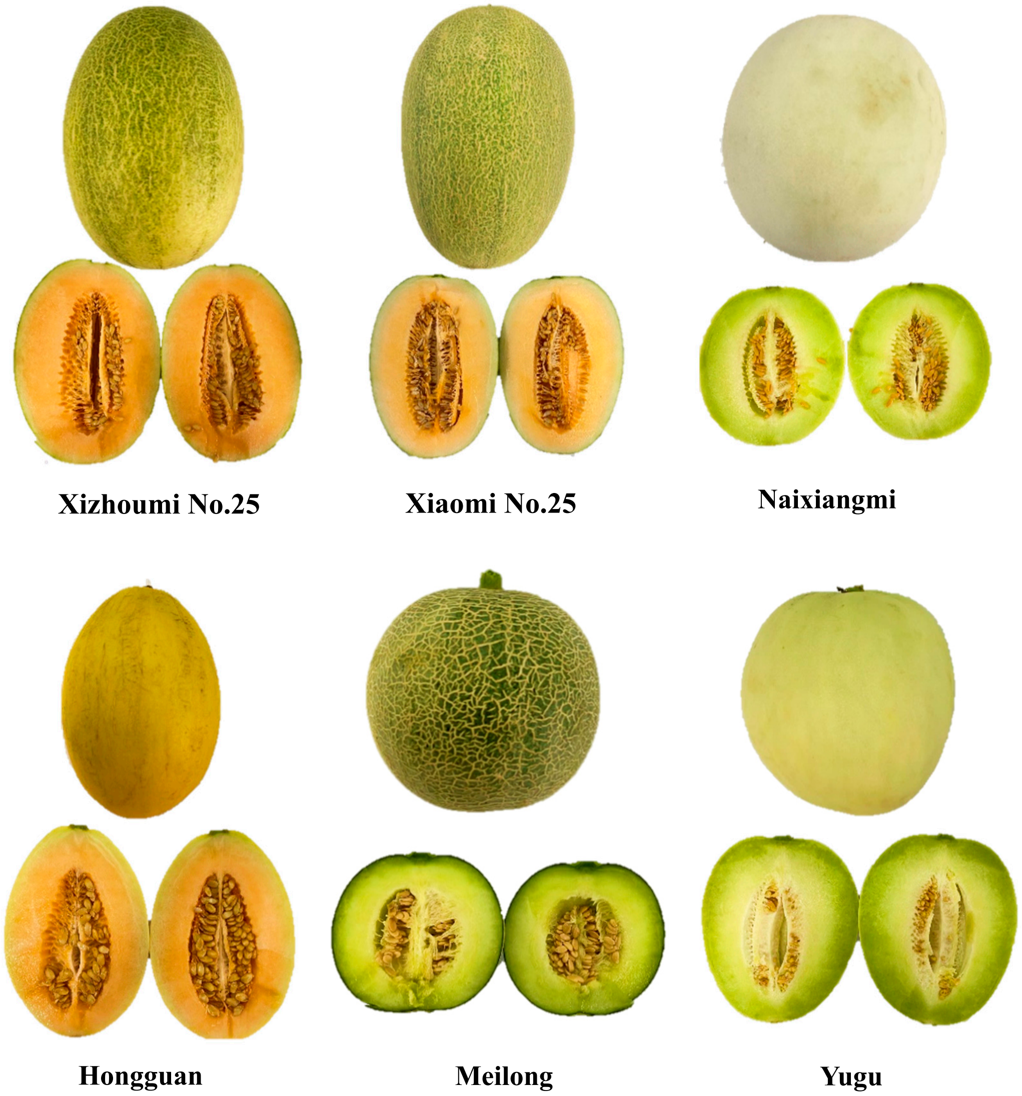

Figure S1 Photographic images of six melon varieties planted in the Hainan province of China.

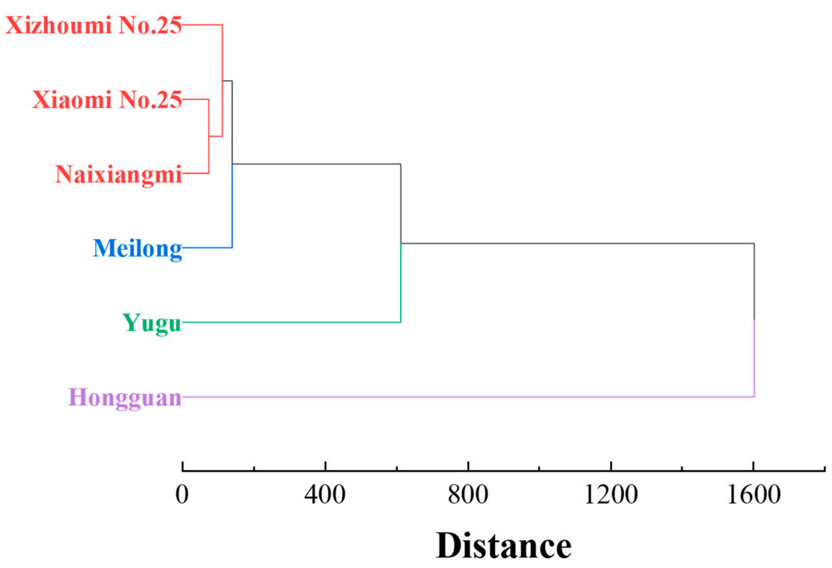

Figure S2 Hierarchical Cluster Analysis of free, bound, and total TPC, FRAP, ABTS^{+}, and phenolic profiles of six melon varieties.

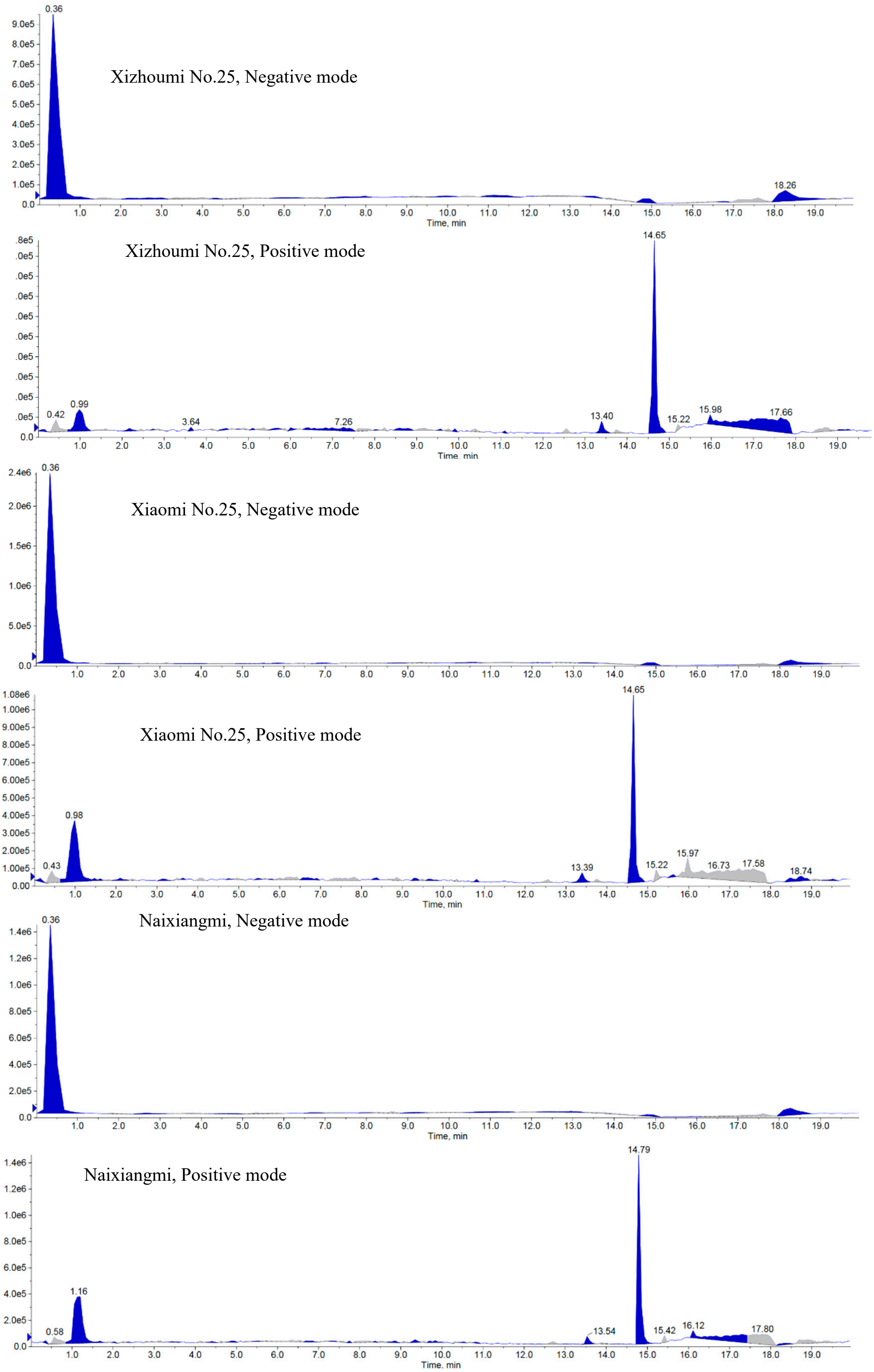
Xizhoumi No.25, Negative mode
0.36
18.26
Time, min
Xizhoumi No.25, Positive mode
0.42
0.99
3.64
7.26
13.40
14.65
15.22
15.98
17.66
Time, min
Xiaomi No.25, Negative mode
0.36
Xiaomi No.25, Positive mode
0.43
0.98
13.39
14.65
15.22
15.97
16.73
17.58
18.74
Time, min
Naixiangmi, Negative mode
0.36
Time, min
Naixiangmi, Positive mode
0.58
1.16
13.54
14.79
15.42
16.12
17.80
Time, min

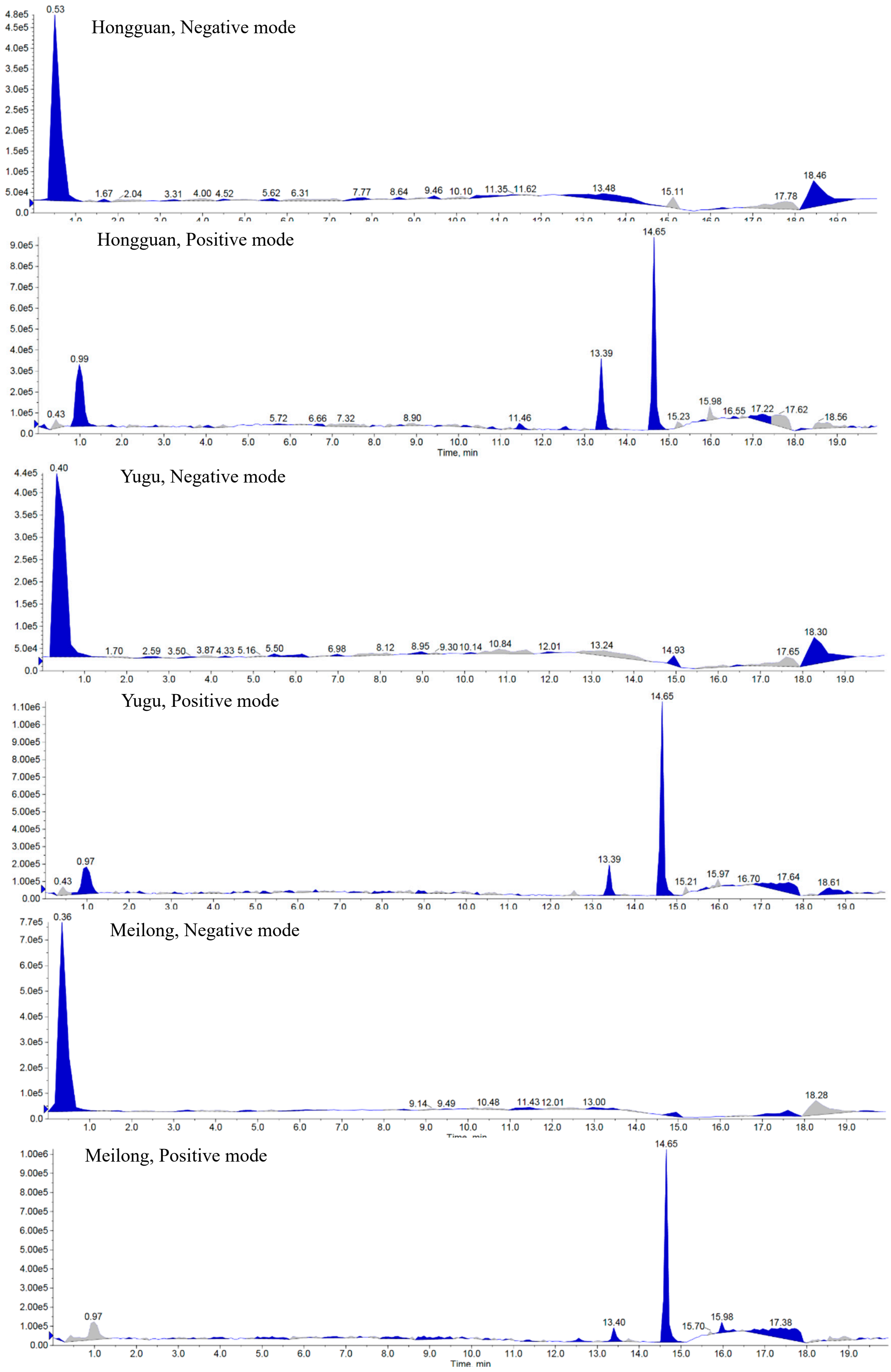

Figure S3 Total ion chromatogram of free extracts of the pulp of six melon varieties analyzed by UHPLC-QQQ-MS in negative and positive modes

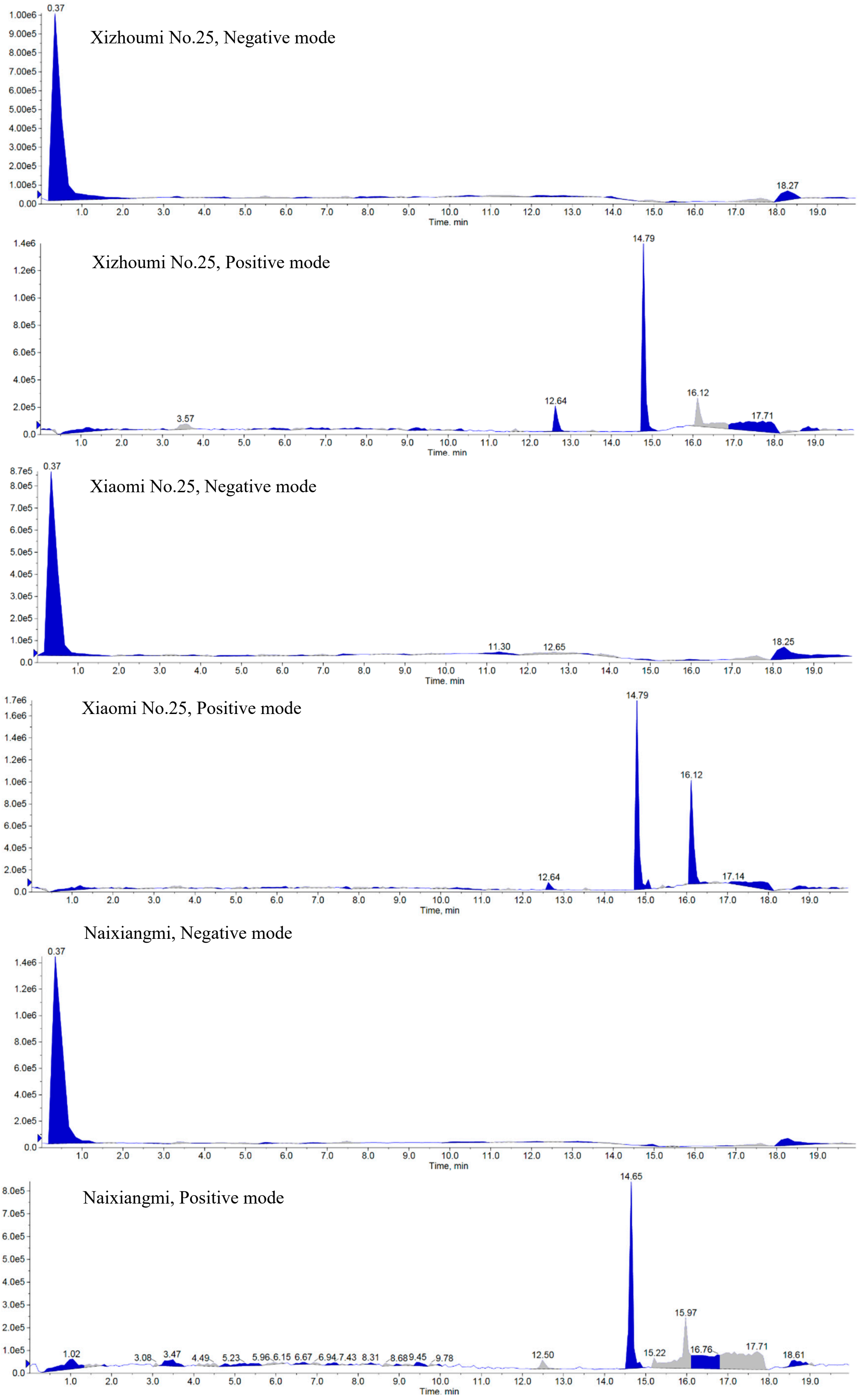
Xizhoumi No.25, Negative mode
0.37
18.27
Time, min
Xizhoumi No.25, Positive mode
3.57
12.64
14.79
16.12
17.71
Time, min
Xiaomi No.25, Negative mode
0.37
11.30
12.65
18.25
Time, min
Xiaomi No.25, Positive mode
12.64
14.79
16.12
17.14
Time, min
Naixiangmi, Negative mode
0.37
Time, min
Naixiangmi, Positive mode
1.02
3.08
3.47
4.49
5.23
5.96
6.15
6.67
6.94
7.43
8.31
8.68
9.45
9.78
12.50
14.65
15.22
15.97
16.76
17.71
18.61
Time, min

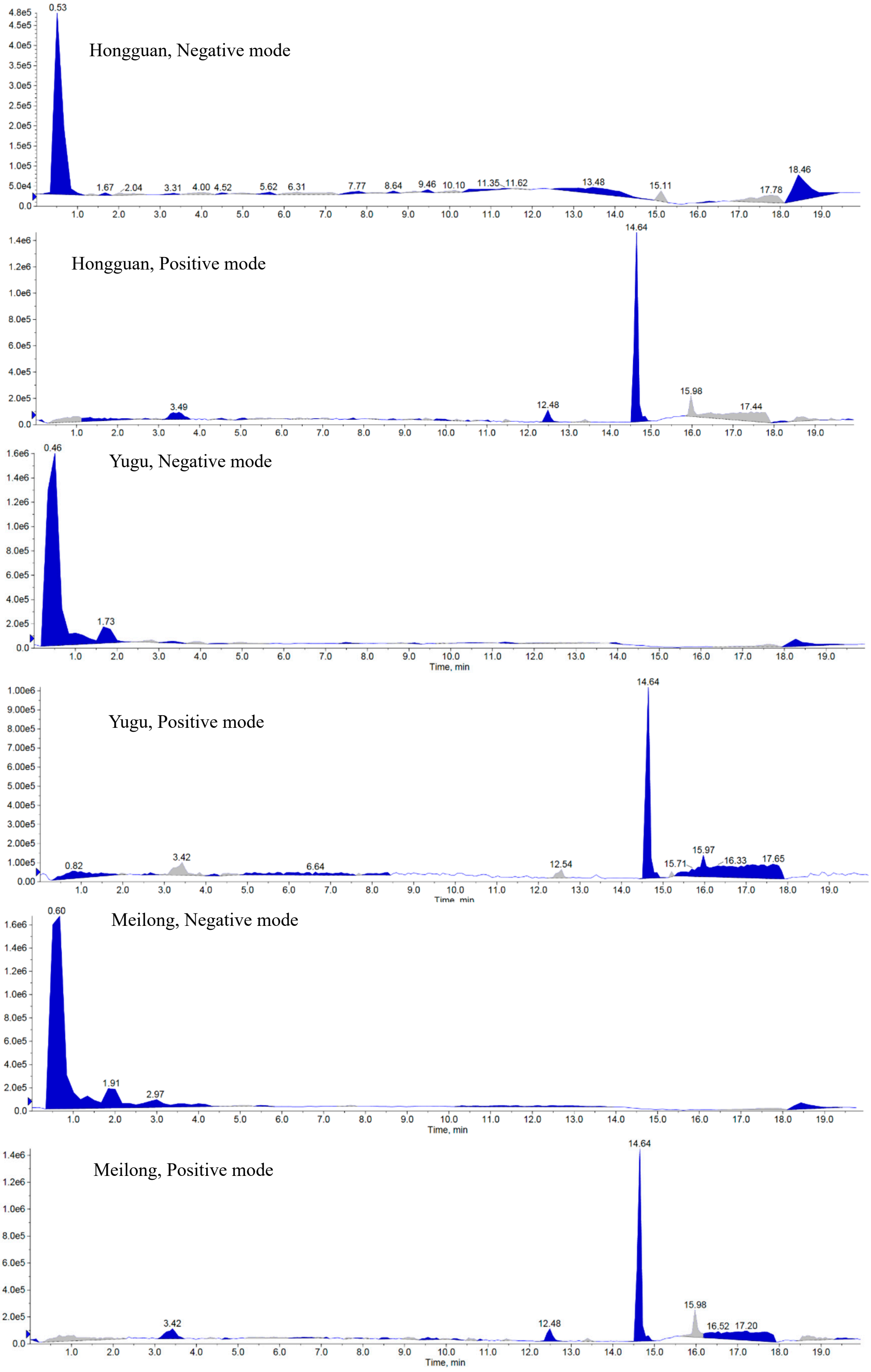

Figure S4 Total ion chromatogram of bound extracts of the pulp of six melon varieties analyzed by UHPLC-QQQ-MS in negative and positive modes.
